# Characteristics of invasive *Staphylococcus aureus* infections in three regions of Korea, 2009-2011: a multi-center cohort study

**DOI:** 10.1186/1471-2334-13-581

**Published:** 2013-12-10

**Authors:** Kyoung-Ho Song, Eu Suk Kim, Hye-yun Sin, Kyung-Hwa Park, Sook-In Jung, Nara Yoon, Dong-Min Kim, Chang Seop Lee, Hee-Chang Jang, Yoonseon Park, Kkot Sil Lee, Yee Gyung Kwak, Jae Hoon Lee, Seong Yeon Park, Minkyo Song, Sue K Park, Yeong-Seon Lee, Hong Bin Kim

**Affiliations:** 1 Department of Internal Medicine, Seoul National University Bundang Hospital, 166 Gumi-ro, Seongnam, Bundang-gu 463-707, Republic of Korea; 2 Department of Internal Medicine, Chonnam National University Hospital, Gwangju, Republic of Korea; 3 Department of Internal Medicine, Chosun University Hospital, Gwangju, Republic of Korea; 4 Department of Internal Medicine, Chonbuk National University Hospital, Jeonju, Republic of Korea; 5 Department of Internal Medicine, Chonnam National University Hwasun Hospital, Hwasun, Jeollanam-do, Republic of Korea; 6 Department of Internal Medicine, National Health Insurance Corporation Ilsan Hospital, Goyang, Republic of Korea; 7 Department of Internal Medicine, Kwandong University Myongji Hospital, Goyang, Republic of Korea; 8 Department of Internal Medicine, Inje University Ilsan Paik Hospital, Goyang, Republic of Korea; 9 Department of Internal Medicine, Wonkwang University Hospital, Iksan, Republic of Korea; 10 Department of Internal Medicine, Dongguk University Ilsan Hospital, Goyang, Republic of Korea; 11 Department of Biomedical Sciences, Seoul National University College of Medicine, Seoul, Republic of Korea; 12 Center for Infectious Diseases, Korea National Institute of Health, Osong, Republic of Korea

**Keywords:** Incidence, Cohort, *Staphylococcus aureus*, Methicillin-resistant, Invasive infection, Bacteremia

## Abstract

**Background:**

Despite the importance of invasive *Staphylococcus aureus* (ISA) infection, its overall burden in non-selected populations has only been defined in a small number of studies in Europe and North America. To define the characteristics of ISA infections in Korea, we conducted a multi-center cohort study to estimate population-based incidence rates.

**Methods:**

We conducted a multicenter prospective cohort study at nine university-affiliated active-surveillance core centers (ASCs) in three regions of Korea. To cover all available clinical microbiologic laboratories, we classified the laboratories in these regions into three groups according to their clinical environment as: 1) Nine ASCs, 2) Five major commercial laboratories and 3) Forty-four acute-care hospital-affiliated microbiology laboratories. We requested all the laboratories to report prospectively their numbers of cases of *S. aureus* isolated from normally sterile sites. Detailed clinical information was collected about the cases in the nine ASCs.

**Results:**

From 1 July 2009 to 30 June 2011, a total of 1,198 cases of ISA infection were identified at the nine ASCs, including 748 (62%) methicillin-resistant *S. aureus* (MRSA) infections. Most (81%) ISA infections were healthcare-associated (HCA): 653 (55%) hospital-onset and 322 (27%) community-onset. 223 (19%) were community-associated infections. The most common primary diagnosis was catheter-associated infection (225 cases, 19%). Respiratory tract infection (160, 13%), skin & soft tissue (152, 13%) and bone & joint infections (120, 10%) were also common. 30-day and 12-week mortality rates were 25.6% (262/1,024) and 36.5% (314/860), respectively. Complications, including metastatic infection within 12 weeks, occurred in 17.8% of ISA infections. The most common site of metastatic infection was the lung (9.8%, 84/860). Based on the total of 2,806 observed cases of ISA infection, estimated annual rates of ISA and invasive MRSA infections were 43.3 and 27.7 per 100,000 populations, respectively.

**Conclusions:**

Our data provide important information about the clinical characteristics of ISA infections. We estimate that over 21,000 ISA infections and 13,000 invasive MRSA infections occurred in Korea in 2010.

## Background

*Staphylococcus aureus* is one of the most important bacterial pathogens. It is not only the major cause of skin and soft tissue infection in the community setting, but also the second most common pathogen causing severe nosocomial infections, including bacteremia, pneumonia and surgical site infections [1]. Invasive *S. aureus* (ISA) infection is associated with a high mortality rate, and imposes a substantial cost and resource burden on the healthcare system [2]. There are also growing concerns about increasing methicillin-resistant *S. aureus* (MRSA) infections in both community and nosocomial settings. Although rates of community-associated (CA) MRSA infection are still low in Korea, MRSA accounts for over 60% of *S. aureus* nosocomial isolates, and there has also been a shift to increasing rates of CA-MRSA disease [3,4].

Despite the importance of ISA infection, its overall burden in non-selected populations has only been defined in a small number of studies in Europe and North America [5-10]. Therefore, we conducted a multi-center prospective cohort study to characterize the clinical spectrum of ISA infections, identify the risk factors for mortality and estimate population-based incidence rates.

## Methods

### Patient population and surveillance

**Figure 1:**
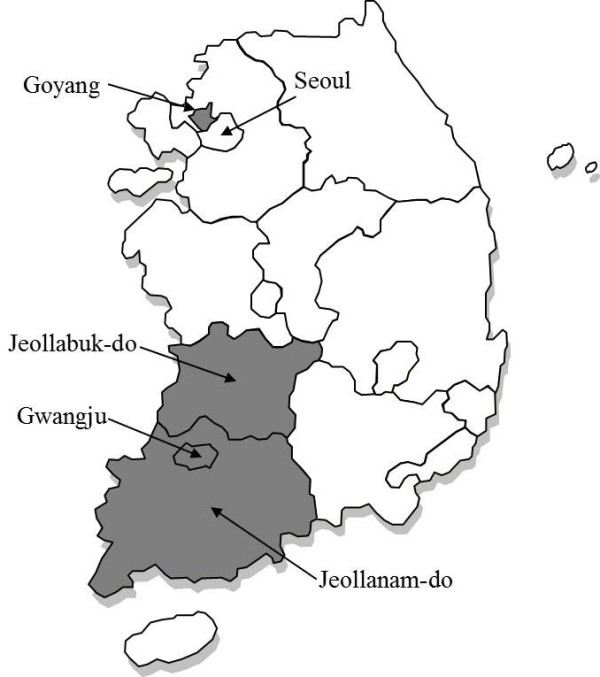
**The three participating Korean regions.** Because of geographical proximity, the 2 provinces (Jeollabuk-do, Jeollanam-do), 1 metropolitan city (Gwangju), and 1 new town (Goyang) were classified into 3 regions (in Gray).

From 1 July 2009 to 30 June 2011, this study was conducted at nine university-affiliated active-surveillance core centers (ASCs), located in two provinces (Jeollabuk-do, Jeollanam-do), one metropolitan city (Gwangju), and one new town (Goyang), which together accounted for 12.1% (5,899,540 / 48,580,293) of the Korean population in 2010 (Figure 1) [11]. Because of their geographical proximity, Gwangju and Jeollanam-do were united into one area, referred to as Gwangju/Jeollanam-do in this study. To estimate population-based incidence rates, we classified the laboratories in the surveillance area into three groups, according to their clinical environment: 1) nine university-affiliated active-surveillance core centers (ASCs), 2) five major commercial laboratories and 3) forty-four acute-care hospital-affiliated microbiology laboratories covering all available clinical microbiologic laboratories.

The nine university-affiliated ASCs (group 1) each had one or more infectious diseases specialists. Cases of *S. aureus* isolated from normally sterile sites were collected prospectively from both the laboratories and the active surveillance core centers by infectious disease specialists. Detailed clinical information about the cases in the nine ASCs was also collected by trained research nurses or infectious diseases specialists using standardized case record forms. All *S. aureus* isolates from normally sterile sites in the 9 ASCs were sent to the coordinating center (Seoul National University Bundang Hospital), to confirm their microbiologic profiles. We also collected the number of cases of *S. aureus* acquired at normally sterile site from all 5 major commercial laboratories, which generally deal with requests from local clinics that lack clinical microbiologic laboratories (group 2). In the end we identified 44 clinical microbiology laboratories qualified by the Korean Association of Quality Assurance for Clinical Laboratories in the surveillance area [12]. We also requested the 44 hospitals (group 3) to report their number of cases of ISA infection. In contrast to the 9 ASCs (group 1), the clinical microbiologic laboratories in group 2 and group 3 were requested to volunteer to participate in this surveillance. In addition, the number of cases was only passively reported without residency data, and separate audits to identify ISA cases were not performed in groups 2 and 3.

This study was approved by the institutional review board of Seoul National University Bundang Hospital in compliance with the Helsinki Declaration.

### Definitions

A case of ISA infection was defined by the isolation of *S. aureus* from a normally sterile site. Normally sterile sites included blood, cerebrospinal fluid, pleural fluid, pericardial fluid, peritoneal fluid, joint/synovial fluid, bone, internal body sites (lymph node, brain, heart, liver, spleen, vitreous fluid, kidney, pancreas, or ovary), and other normally sterile sites. When repeated cultures from normally sterile sites were positive in a single patient within 12 weeks of the onset of illness, the number of ISA infections was counted as one.

Community-onset infections were those that were either present or incubating at the time of hospital admission, or were found in the first positive culture obtained within 48 h of admission. Community-onset infections were classified into healthcare-associated (HCA) and community-associated (CA). If a patient fulfilled any of the following criteria, the infection was classified as HCA [9]: (1) presence of an invasive device at the time of admission or of onset of infection; (2) history of MRSA infection or colonization in the previous 6 months; (3) history of surgery, hospitalization, dialysis, or residence in a long-term care facility in the previous 12 months. ISA infection was classified as CA when it did not fit the above criteria for HCA infection. A case was classified as hospital-onset if the first *S. aureus* culture was obtained 48 h or more after admission to the hospital. A primary diagnosis was made on the basis of the clinical, radiological, and microbiological information [13]. Clinical isolates were confirmed as *S. aureus*, and were tested for antimicrobial susceptibility by standard techniques. The antimicrobial therapy was considered ‘appropriate’ if the treatment regimen included antibiotics active in vitro, and the dosage and route of administration were in conformity with current medical standards. Metastatic infection was defined as the presence of microbiological or radiographic evidence of *S. aureus* infection caused by hematogenous seeding [14]. The initial treatment response was assessed 72 hours after starting antimicrobial therapy, and ‘failure’ was defined if patients died or when their illness deteriorated or there was no reduction in fever, leukocytosis or other signs of infection [15].

### Data management and analyses

Differences in proportions were compared by Fisher’s exact test or the chi-square test, and means were compared by Student’s *t*-test. To identify the independent risk factors for 30-day mortality, a stepwise multiple logistic regression model was used. Risk factors with *P* values of less than .10 in univariate analysis were included in the initial model, and forward stepwise selection was performed to define significant risk factors. Charlson’s weighted index of co-morbidity (WIC) and all underlying diseases were analyzed in a separate logistic regression model to avoid data overlap in the multivariate analysis. *P* values of less than .05 were considered statistically significant in the multivariate analysis. IBM PASW for Windows (*version* 20 software package; SPSS Inc., Chicago, IL, USA) was used for all analyses.

## Results

### Clinical characteristics of ISA infections

During the 2-year period (1 July 2009 to 30 June 2011), a total of 1,198 cases of ISA infections, including 748 (62%) MRSA infections, were observed at 9 ASCs. Median age of patients was 64 years (interquartile range, IQR: 48 to 74 years), and 733 (61%) patients were male. Most (81%) ISA infections were HCA infections: 653 (55%) hospital-onset, 322 (27%) community-onset. 223 (19%) were CA infections. Of 1,198 cases, 1,026 (86%) patients had ≥1 positive blood-culture result, and 38 (3%) had a positive result for pleural fluid. Peritoneal fluid yielded positive results for 37 patients (3%), CSF for 26 (2%), synovial fluid for 23 (2%), culture of deep-tissue samples for 22 (2%) and other sterile sites for 26 (2%).

Excluding the cases with unknown primary site of infection, the most common primary diagnosis was catheter-associated infection (225 cases, 19%). Respiratory tract infection, skin & soft tissue and bone & joint infections were also common. The clinical spectra and characteristics of the ISA infections according to site of infection are listed in Table 1.

**Table 1:** **Clinical characteristics of 1,198 patients with invasive *Staphylococcus aureus *** **infections in 9 active surveillance core centers in 3 regions of Korea, July 2009 ~ June 2011**

**Primary diagnosis, infection**	**No. of Cases**	**No. of MRSA infection**	**Age, median in year (IQR)**	**Community-associated**	**Healthcare-associated**		**30–day mortality, n/N (%)**
					**Community-onset**	**Hospital-onset**	
Catheter-associated	225 (19)	169 (75)	63 (49-63)	0	57 (25)	168 (75)	52/201 (26)
Respiratory tract	160 (13)	109 (68)	70 (55-77)	34 (21)	32 (20)	94 (59)	52/132 (39)
Skin and soft tissue	152 (13)	71 (47)	65 (51-74)	40 (26)	50 (33)	62 (41)	23/128 (18)
Surgical site	103 (9)	87 (85)	61 (45-70)	6 (6)	17 (17)	80 (78)	13/99 (13)
Bone and joint	120 (10)	57 (48)	67 (54-73)	51 (43)	37 (31)	32 (27)	14/107 (13)
Endocarditis	23 (2)	7 (30)	64 (45-72)	9 (39)	13 (57)	1 (4)	7/18 (39)
Intra-abdominal	84 (7)	62 (74)	64 (45-74)	12 (14)	30 (36)	42 (50)	21/76 (28)
Urinary tract	22 (2)	13 (59)	69 (56-76)	5 (23)	7 (32)	10 (46)	6/17 (35)
Others	4 (0)	4 (100)	50 (39-60)	1 (25)	1 (25)	2 (50)	0/3 (0)
Unknown	305 (26)	169 (55)	62 (38-74)	65 (21)	78 (26)	162 (53)	74/243 (30)
Total	1,198	748 (62)	64 (48-74)	223 (19)	322 (27)	653 (55)	262/1,024 (26)

Data indicate no. (%) of patients. MRSA, methicillin-resistant *S. aureus*; IQR, interquartile range.

### Clinical outcomes and risk factors for mortality

The 30-day mortality rate was 25.6% (262/1,024). 12-week mortality rate was 36.5% (314/860). 455 (52.9%) patients recovered from ISA infection without recurrence or complications, assessed at 12 weeks after onset of illness. 3.1% (27/860) of the patients with ISA infections suffered recurrence within 12 weeks, and 16% (138/860) had metastatic infections. The most common site of metastatic infection was the lung (9.8%, 84/860). The clinical outcomes of the ISA infections are listed in Table 2.

**Table 2:** **Clinical outcomes of invasive *Staphylococcus aureus *** **infections in 9 active surveillance core centers in 3 regions of Korea, July 2009 ~ June 2011**

**Patients with**	**No. of cases (%)**
Treatment failure assessed at 72 hours after onset of illness	225 /991 (22.7)
30-day mortality	262 /1,024 (25.6)
Clinical outcomes assessed at 12-week after onset of illness	Total No. = 860
Recurrence	27 (3.1)
Complications	153 (17.8)
Metastatic infections (site of metastasis)^a^	138 (16.0)
Heart^b^	20 (2.3)
Lung	84 (9.8)
Bone^c^	8 (0.9)
Fundus^d^	6 (0.7)
Skin	18 (2.1)
Others^e^	20 (2.3)
Survival without recurrence or complications	455 (52.9)
12-week mortality	314 (36.5)

Data indicate no. (%) of patients.

^a^15 patients were diagnosed with 2 or more sites of metastatic infection.

^b^Echocardiography was performed in 319 patients.

^c^Bone scan and/or MRI was performed in 82 patients.

^d^Ophthalmologic examination was performed in 53 patients.

^e^Others: these are central nervous system (4 patients), intra-abdominal (6), joint space (6), myositis (2), pericardial (1), and endovascular (1) infections.

The significant risk factors associated with 30-day mortality identified by univariate analysis, and the results of multivariate analysis of risk factors, are presented in Table 3. In multivariate analysis, extreme age (≥65 or <6 years), steroid use within 1 month, severe underlying disease (Charlson’s WIC ≥3), presentation with severe sepsis or septic shock, presence of metastatic infection of the lung, and inappropriate empirical antibiotics were significantly associated with 30-day mortality due to ISA infection. On the other hand, skin and soft tissue infection decreased the risk of 30-day mortality. Among the underlying illnesses, connective tissue disease, moderate to severe liver disease, and metastatic solid tumor were independently associated with 30-day mortality.

**Table 3:** **Significant risk factors associated with 30-day mortality in 1,024 patients with invasive *Staphylococcus aureus *** **infections in 9 active surveillance core centers in 3 regions of Korea, July 2009 ~ June 2011**

**Risk factors**	**No. of Survivors (N = 762)**	**No. of Non-survivors (N = 262)**	**Univariate OR (95% CI)**	**Multivariate OR**^**a **^**(95% CI)**
Age, ≥65 or <6 years	370 (49)	181 (69)	2.367 (1.757-3.189)	2.068 (1.822-3.575)
Steroid use within 1 month	67 (9)	40 (15)	1.869 (1.228-2.844)	1.891 (1.150-3.107)
Indwelling medical device	287/741 (39)	112/248 (45)	1.303 (0.974-1.742)	NS
Long-term care facility resident	288/703 (41)	117/240 (49)	1.371 (1.021-1.840)	NS
Underlying illness				
Charlson’s WIC, ≥3	235 (31)	130 (50)	2.209 (1.658-2.942)	1.796 (1.294-2.495)
Chronic pulmonary disease	26 (3)	23 (9)	2.724 (1.526-4.864)	NS
Connective tissue disease	20 (3)	14 (5)	2.094 (1.042-4.209)	3.042 (1.342-6.895)
Ulcer disease	71 (9)	41 (16)	1.806 (1.194-2.729)	NS
Moderate to severe liver disease	36 (5)	30 (12)	2.608 (1.571-4.328)	4.004 (2.205-7.273)
Metastatic solid tumor	31 (4)	38 (15)	4.000 (2.433-6.578)	3.724 (2.106-6.584)
Primary diagnosis, infection				
Respiratory tract	80 (10)	52 (39)	2.111 (1.441-3.093)	NS
Skin and soft tissue	105 (14)	23 (9)	0.602 (0.375-0.968)	0.598 (0.340-0.998)
Surgical site	86 (11)	13 (5)	0.410 (0.225-0.749)	NS
Bone and joint	93 (12)	14 (5)	0.406 (0.227-0.726)	NS
Unknown	169 (22)	74 (28)	1.381 (1.004-1.900)	NS
MRSA infection	467 (61)	181 (69)	1.412 (1.046-1.905)	NS
Severe sepsis or septic shock	65 (9)	88 (34)	5.423 (3.780-7.781)	6.173 (4.107-9.277)
Metastatic infection of lung	46 (6)	27 (10)	1.788 (1.087-2.941)	2.169 (1.223-3.847)
Inappropriate empirical antibiotics	323 (42)	129 (49)	1.318 (0.995-1.747)	NS
Inappropriate definitive antibiotics	85 /756 (11)	35 /214 (17)	1.650 (1.084-2.512)	3.199 (2.352-4.350)

Data indicate no. (%) of patients. Total no. of cases was 1,024, because detailed clinical information was not available for 174 patients. Charlson’s weighted index of co-morbidity and each underlying diseases were analyzed in separate logistic regression models to avoid data overlap in the multivariate analysis.

OR, odds ratio; CI, confidence interval; MRSA, methicillin-resistant *S. aureus*; Charlson’s WIC, Charlson’s weighted index of co-morbidity; NS, not significant.

^a^Risk factors with *P* values of less than .10 in univariate analysis were included in the initial model. *P* values of less than .05 were considered statistically significant in the multivariate analysis.

### Estimating incidence rates

In addition to the 1,198 cases of ISA infection that were identified by the 9 ASCs, 724 were reported from the 5 major commercial laboratories. Of the total of 44 hospitals with clinical microbiology laboratories, 10 (23%) volunteered to respond to our approach, and reported 884 cases of ISA infections. Thus during the 2-year period, a total of 2,806 cases of ISA infection were recorded by surveillance, including 1,820 (64.9%) MRSA infections. Median numbers of beds in the participating 10 hospitals and 34 non-participating ones were 257 (interquartile range, IQR: 201-493) and 235 (IQR: 193-294), respectively (*P* = 0.303 by *t*-test). After adjusting by the number of total beds in the 44 hospitals, we estimated the number of ISA infections in those hospitals to be 3,170.

The crude annual rates of ISA and invasive MRSA infections in the 3 regions of Korea in 2010 were estimated to be 43.3 and 27.7 per 100,000 populations, respectively. Estimated incidences of ISA infection, *S. aureus* bacteremia, invasive MRSA infection and MRSA bacteremia by surveillance site are listed in Table 4.

**Table 4:** **Estimated incidence of invasive *Staphylococcus aureus *** **infection in 3 regions of Korea, July 2009 ~ June 2011**

**Surveillance site**	**Total estimated No. of cases (per year)**	**Incidence per 100,000 person-year**				**Total population in 2010**
		**MRSA bacteremia**	**All *S. aureus *** **bacteremia**	**Invasive MRSA infection**	**All invasive *S. aureus *** **infection**	
Goyang	180	8.9	15.2	11.2	19.9	905,076
Jeollabuk-do	552	15.2	21.7	21.8	31.0	1,777,220
Gwangju/Jeollanam-do	1,824	29.2	46.7	35.6	56.7	3,217,244
Total	2,555	21.9	34.4	27.7	43.3	5,899,540

MRSA, methicillin-resistant *S. aureus.*

## Discussion

In the present study we described the clinical characteristics of ISA infection in Korea based on multicenter cohort data. Excluding the cases of unknown primary site of infection, the most common primary diagnosis was catheter-associated infection (225 cases, 19%), followed by pneumonia and skin and soft tissue infection. Because we gathered the detailed clinical information from only 9 ASCs (group 1), which were mostly tertiary-care and/or university-affiliated hospitals, our data has a tendency to over-represent healthcare-associated settings. In fact, 975 (81%) cases were healthcare-associated. Hence, we cannot extrapolate the observed clinical characteristics to all community-based institutions. However, considering that there are few epidemiologic data for Korea, we believe that our findings on the clinical spectra should provide valuable information about ISA infection.

We followed up 72% (860/1,198) of the patients for 12 weeks to evaluate recurrence and complications, as well as mortality. 12 week mortality was quite high (36.5%) and survival without recurrence or complications was only 52.9%. Complications including metastatic infection occurred in 17.8% of the ISA infections, similar to previous reports [1,16]. However, subsequent endocarditis occurred in only 2.3% of our patients. Considering that 86% of the patients had ≥1 positive blood-culture result, endocarditis was quite rare compared to previous studies of *S. aureus* bacteremia [17-19]. However, we should take into account that echocardiography was performed in only 319 (37%) patients, and transesophageal echocardiography in only 20 (2.3%) patients. Therefore, to evaluate accurately the prevalence of endocarditis after ISA infection, further studies are needed using universal or selected screening echocardiography, especially in *S. aureus* bacteremic patients.

A number of conditions were identified as risk factors for 30-day mortality in patients with ISA infection in the nine ASCs (Table 4). As in previous studies of *S. aureus* bacteremia, older age, use of immunosuppressants, severe underlying disease, high severity of illness, and inappropriate definitive antibiotics were independent risk factors for mortality [8,20-22]. In contrast to other studies, respiratory tract infection itself did not increase the risk of death, whereas metastatic infection of the lung was an independent risk factor [21,22]. However, because there was a possibility of misclassification between the primary focus and metastatic infection site, the clinical impact of this finding is unclear. Meanwhile, a number of studies have suggested that methicillin resistance is a risk factor for mortality in patients with *S. aureus* infection [23,24]. However, in our study, MRSA infection was not associated with increased risk of 30-day mortality in multivariate analysis.

In the present study we tried to estimate incidence rates of ISA infection in Korea for the first time. Based on the 2,806 observed cases of ISA infection, we estimated that over 21,000 ISA infections and 13,000 invasive MRSA infections occurred in Korea in 2010. Our estimated incidences of ISA and invasive MRSA infection (43.3 and 27.7 per 100,000 populations) are higher than those in Calgary, Canada and comparable to those in the United States [8,9,20]. Recently, Laupland *et al.* reported that the overall contemporary incidence of *S. aureus* bloodstream infection (SA-BSI) has averaged 26.1 per 100,000 in 9 regions of 5 countries; Finland, Australia, Sweden, Canada and Denmark [25]. Population-based studies conducted in many regions around the world have identified SA-BSI incidence rates of 15–40 per 100,000 population per year [6,8,20,26-28]. Our estimate (*S. aureus* bacteremia: 34.4 per 100,000, interval 15.2-46.7) was thus similar to, or somewhat higher than, that of other countries.

Patients who live outside a given population area frequently have characteristics that are different from those of the endogenous population, and failure to exclude these cases will result in an overestimate of disease occurrence as well as introduce a ‘referral bias’ [29]. Among the total of 1,510 ISA infections, we excluded 312 (20.7%) patients who were not resident in the studied population of the 9 ASCs (group 1). However, we did not investigate where the patient in group 2 and group 3 resided. However, considering that the ISA cases in groups 2 and 3 came from community-based clinics and hospitals with acute presentation and severe ISA infections, we believe that the effect of imported cases was minimal. On the other hand, we may actually have underestimated the incidence of invasive MRSA disease if persons in the surveillance areas sought health care from facilities using laboratories outside that area. In our data, the incidence (19.9 per 100,000) of ISA infection in Goyang was only one-third of that (56.7) in Gwangju/Jeollanam-do. This difference may have been due to the proximity of Goyang to the Seoul metropolitan area (Figure 1). According to “Medical service usage statistics per region in 2010” produced by the National Health Insurance Corporation, the rates of internal use of medical treatment facilities (medical treatment facilities in the registered region divided by the total facilities) were 88.4% in Jeollabuk-do and 82.2% in Gwangju/Jeollanam-do, but only 56.1% in Goyang (*cf.* 90.4% in Seoul) [30]. Thus, we think that the actual incidence of ISA infections in the 3 regions was higher than our estimate.

The measured incidence of invasive infection is dependent on the number of diagnostic tests performed. Unfortunately, we did not collect data on how many blood cultures and invasive diagnostic samples were tested at each hospital. However, Kim *et al.* recently reported the number of blood cultures examined per 1,000 patient days at seven Korean university-affiliated hospitals in 2010 [31]. Because the characteristics of the 7 participating university-affiliated hospitals were similar to those of our 9 ASCs, we could assume that our number of blood cultures was similar. The mean number of blood cultures per 1,000 days was 110 ±23 (SD), range 65 to 129 [31], which is similar to or somewhat lower than the number (103-188) of blood cultures per 1,000 patient days suggested by Cumitec 1C [32]. The difference might be secondary to a difference in the proportion of patients with acute illnesses or to different blood culture ordering systems. Most hospitals (about 70%) in Korea examine two blood cultures per adult patient and one culture per child patient (about 80%) rather than three sets.

Although we tried to evaluate all cases of ISA infection in the three Korean regions, we were unable to collect ISA cases from a large number (34) of the 44 hospitals in group 3. While there was no significant difference in number of beds between the 10 participating hospitals and other 34 hospitals, our group 3 data could be biased because we depended on voluntary reporting of numbers of ISA infections by these hospitals (i.e. volunteer bias). Nevertheless, because these were community-based hospitals whose patients have similar clinical characteristics, we were obliged to estimate the rate of ISA infection in the 44 hospitals from the cases in the 10 hospitals that responded to our approach, based on the difference in number of beds. When we calculated the incidence from the exact number of cases observed, the crude annual rates of ISA and invasive MRSA infections were reduced to 23.8 (18.9 ~ 27.8) and 15.4 (11.2 ~ 17.8) per 100,000 populations, respectively.

## Conclusions

In conclusion, this is the first large-scale prospective cohort study to evaluate the clinical characteristics of patients with ISA infections and the risk factors for mortality, together with an attempt to estimate population-based incidence rates. We have estimated that over 21,000 ISA infections and 13,000 invasive MRSA infections occurred in Korea in 2010. We believe that further studies based on well-documented, non-selected populations are warranted in order to establish healthcare and research priorities and evaluate the effectiveness of preventative interventions.

## Competing interests

This work was supported by a grant from the Korea Centers for Disease Control and Prevention. All authors declare that they do not have any competing interests.

## Authors’ contributions

KHS, ESK, and HBK participated in the design of the study, analyzed data, and drafted the manuscript. KHP, SIJ, NY, DMK, CSL, HCJ, YP, KSL, YGK, JHL, and SYP participated in the design of the study and collected the cases of ISA infection. KHS, HYS, YSL, and HBK coordinated clinical data collection and management. MS and SKP helped with the statistical analysis. All authors were involved in interpreting the data and critical revision of the manuscript, and all read and approved the final manuscript.

## Pre-publication history

The pre-publication history for this paper can be accessed here:

http://www.biomedcentral.com/1471-2334/13/581/prepub
